# Prenatal Cannabinoid Exposure Elicits Memory Deficits Associated with Reduced PSA-NCAM Expression, Altered Glutamatergic Signaling, and Adaptations in Hippocampal Synaptic Plasticity

**DOI:** 10.3390/cells12212525

**Published:** 2023-10-26

**Authors:** Priyanka D. Pinky, Jenna Bloemer, Warren D. Smith, Yifeng Du, Ryan T. Heslin, Sharay E. Setti, Jeremiah C. Pfitzer, Kawsar Chowdhury, Hao Hong, Subhrajit Bhattacharya, Muralikrishnan Dhanasekaran, Alexander Dityatev, Miranda N. Reed, Vishnu Suppiramaniam

**Affiliations:** 1 Department of Drug Discovery and Development, Auburn University, Auburn, AL 36849, USA; 2 Department of Biomedical Engineering, University of California Irvine, Irvine, CA 92697, USA; 3 Department of Pharmaceutical and Biomedical Sciences, Touro College of Pharmacy, New York, NY 10036, USA; 4 Key Laboratory of Neuropsychiatric Diseases, Jiangsu Key Laboratory of Drug Discovery for Metabolic Diseases, and State Key Laboratory of Natural Medicines, China Pharmaceutical University, Nanjing 210009, China; 5 Center for Neuroscience Initiative, Auburn University, Auburn, AL 36849, USA; 6 Keck Graduate Institute, School of Pharmacy and Health Sciences, Claremont Colleges, Claremont, CA 91711, USA; 7 Molecular Neuroplasticity, German Center for Neurodegenerative Diseases (DZNE), 37075 Magdeburg, Germany; 8 Medical Faculty, Otto-von-Guericke University, 39106 Magdeburg, Germany

**Keywords:** prenatal, cannabinoid, memory, behavior, developmental, glutamate, synaptic plasticity, marijuana, adolescence

## Abstract

Cannabis is now one of the most commonly used illicit substances among pregnant women. This is particularly concerning since developmental exposure to cannabinoids can elicit enduring neurofunctional and cognitive alterations. This study investigates the mechanisms of learning and memory deficits resulting from prenatal cannabinoid exposure (PCE) in adolescent offspring. The synthetic cannabinoid agonist WIN55,212-2 was administered to pregnant rats, and a series of behavioral, electrophysiological, and immunochemical studies were performed to identify potential mechanisms of memory deficits in the adolescent offspring. Hippocampal-dependent memory deficits in adolescent PCE animals were associated with decreased long-term potentiation (LTP) and enhanced long-term depression (LTD) at hippocampal Schaffer collateral-CA1 synapses, as well as an imbalance between GluN2A- and GluN2B-mediated signaling. Moreover, PCE reduced gene and protein expression of neural cell adhesion molecule (NCAM) and polysialylated-NCAM (PSA-NCAM), which are critical for GluN2A and GluN2B signaling balance. Administration of exogenous PSA abrogated the LTP deficits observed in PCE animals, suggesting PSA mediated alterations in GluN2A- and GluN2B- signaling pathways may be responsible for the impaired hippocampal synaptic plasticity resulting from PCE. These findings enhance our current understanding of how PCE affects memory and how this process can be manipulated for future therapeutic purposes.

## 1. Introduction

Cannabis is one of the most commonly used illicit drugs worldwide. Cannabis use has increased in the American continent in the past decade from 6.9% to 8% of the adult population [1]. Its use during pregnancy has also increased dramatically in the past ten years [2], and the recent relaxation of marijuana policies in several countries is expected to result in even greater maternal use [3]. The major psychoactive ingredient in marijuana and other cannabis-based preparations is Δ9-tetrahydrocannabinol (THC), which can cross the placental barrier to expose the fetus [4,5]. As the prenatal brain is particularly sensitive to maternal drug use, prenatal cannabinoid exposure (PCE) has been demonstrated to cause behavioral and cognitive alterations in the offspring [6,7,8,9].

The cannabinoid receptor type 1 (CB_1_R) is expressed early during prenatal brain development and is functionally coupled to signal transduction mechanisms from early prenatal stages [10,11]. Given that CB_1_R plays an important role in CNS development, affecting synaptogenesis, proliferation, and migration of neuronal cells, functional synaptic organization, and signal transduction (as reviewed in [12]), persistent cognitive deficits after PCE are not surprising. While there are recent studies demonstrating the long-term consequences of pre- and peri-natal cannabinoid exposure [13,14,15,16,17], there is, nevertheless, a relative paucity of literature compared to other drugs of abuse regarding the mechanisms mediating cognitive deficits that result from PCE.

In the current study, we used a rodent model to determine the long-lasting consequences of PCE on hippocampal glutamatergic neurotransmission and memory during adolescence. We also identified a potential mechanism for these deficits, notably a reduction in polysialylated-neural cell adhesion molecule (PSA-NCAM). NCAM is a transmembrane glycoprotein essential for maintaining neurite outgrowth, cell migration, and synaptogenesis. PSA, a highly negatively charged homomeric polymer of sialic acid, is attached to the extracellular domain of NCAM and greatly affects NCAM function [18,19,20]. We have previously shown that perturbation in either polysialylation or NCAM expression causes deficits in hippocampal long-term potentiation (LTP) and hippocampal-dependent memory tasks [19], and these alterations can be rescued by blocking extrasynaptic GluN2B activity [18]. Together with our current findings, we propose that PCE may lead to alterations in glutamatergic synaptic plasticity through reduction in PSA-NCAM expression, ultimately leading to memory deficits. 

## 2. Materials and Methods

### 2.1. Animals

Timed pregnant Sprague Dawley rats were purchased from Envigo laboratories. Pregnant dams were anaesthetized by using isoflurane (4% flow in oxygen) on gestational day 3 (GD 3) to implant an osmotic minipump (Alzet, 2004) subcutaneously. The isoflurane flow was lowered to 1.5–3% in oxygen, and proper heating was ensured to maintain the body temperature. The pump was filled with either vehicle (N-methyl-2-pyrrolidone) or the cannabinoid agonist WIN55,212-2 (Sigma-Aldrich, Burlington, MA, USA) at 2 mg/kg body weight/day [21,22,23,24]. The pumps were removed on postnatal day 2 (PND 2), and male offspring were weaned at PND 21. This dose is not associated with maternal or fetal abnormalities, including no alterations in maternal weight, fetal weight, litter size, gestation time, or pup mortality [25,26,27]. Likewise, our data show no differences in pup mortality, litter sizes, the day of fur development and eye opening, righting reflex on days 5, 8, or 11, or body weight. Experimental procedures were performed between PND 40–65 days. Animals were housed in a vivarium maintained on a 12 h:12 h light: dark cycle. All procedures were carried out in accordance with NIH guidelines and approved by Auburn University Animal Care and Use Committee (IACUC). Sample size calculation, study design, statistical analysis, result reporting, etc., were performed following ARRIVE guidelines. 

### 2.2. Experimental Timeline 

**Figure 1:**
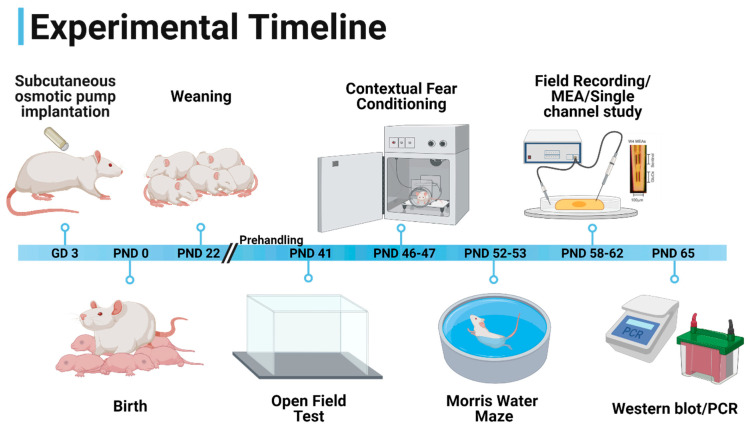
Experimental timeline. Subcutaneous osmotic minipump implantation was performed on gestational day (GD) 3. WIN55,212-2 or vehicle were delivered until the birth of the pups. Pups were weaned on postnatal day (PND) 21. Animals were handled for 5 days prior to the behavioral experiments. Open field test, contextual fear conditioning, and Morris water maze were performed at PND 41, PND 46–47, and PND 52–53. Behaviorally characterized animals were then divided amongst the following: electrophysiological experiments or in vivo glutamate recordings (PND 58–62) or Western blotting and PCR studies (PND 65). This figure was “Created with BioRender.com” (https://biorender.com/ accessed on 3 December 2021).

Prior to behavioral testing, animals were acclimated to transport and handling by pre-handling for 5 days. Pre-handling consisted of daily weighing and 5 min of gentle handling while walking. All the behavioral experiments were performed in the same group of male animals, starting with the open field test on PND 41 and followed by contextual fear conditioning on PND 46–47 and the Morris water maze on PND 52–53. Rats were used for either electrophysiological experiments or in vivo glutamate recordings on PND 58–62, or immunoblotting and PCR experiments on PND 65, as shown in Figure 1.

### 2.3. Behavioral Testing

#### 2.3.1. Open Field 

The open field test was performed to assess the general locomotor ability of the rats. Each rat was placed in a transparent 60 × 60 cm plexiglass arena and allowed to explore for 10 min, after which the rat was returned to its home cage. The trial was recorded via a camera and EthoVision XT 11.5 software was used to track the rat. A virtual center square (45 × 45 cm) was defined as the “center zone”, whereas the remainder was defined as the “outer zone”. Anxiety parameters included the time (s) spent in, and number of entries into, the center zone of the arena. Activity measures included mean speed (cm/s) and distance traveled (cm) in the whole arena [28,29]. 

#### 2.3.2. Contextual Fear Conditioning

The contextual fear conditioning procedure was conducted over 2 days to assess hippocampal-dependent memory. On day 1, animals were placed in the conditioning chamber, and, 180 s later, a single 2 s, 0.75 mA foot-shock was delivered [30]. The program measured freezing behavior, defined as the absence of motion except that required for respiration, for 1 s. The software calculated the percent time freezing using data from the near infrared camera. Animals were removed from the context 30 s after foot-shock delivery. Freezing behavior prior to the onset of the shock was used to compare baseline levels of freezing among the groups. On day 2, 24 h later, the animals were returned to the training context and exposed to all contextual stimuli, but not the shock, for 180 s. Freezing behavior during the 180 s period was used to compare contextual fear memory among the groups. 

#### 2.3.3. Morris Water Maze

Morris water maze (MWM) was performed next to assess hippocampal-dependent spatial memory. The rapid acquisition version of MWM was performed as previously described [31,32]. The rats received eight training trials, in which they were released into a pool filled with black colored water from four different start locations (North, South, East, West). Animals were allotted 60 s to find the submerged platform, which was located 2 cm beneath the water surface. For the rapid acquisition training, pathlength (distance to locate the platform in cm) was compared between groups. To assess hippocampal-dependent spatial reference memory, a probe trial was conducted 24 h after the last training session. For the probe trial, the platform was removed, and animals were placed in the pool for 60 s. Percent time spent in the target quadrant versus the average of the other three quadrants was compared within and between groups. 

### 2.4. Electrophysiological Recording

#### 2.4.1. Preparations of Acute Hippocampal Slices

For electrophysiological experiments, transverse hippocampal slices (350 μm) from male pups were prepared following euthanasia. Euthanasia was performed in accordance with American Veterinary Medical Association (AVMA) guidelines 2013 edition. Animals were placed in the chamber one at a time, followed by the addition of CO_2_ from a compressed gas cylinder at a low flow rate. Animal death was verified before removing from the chamber. Following decapitation, hippocampal slices were sectioned submerging in ice cold cutting buffer containing (in mM) 85 NaCl, 2.5 KCl, 4.0 MgSO_4_, 0.5 CaCl_2_, 1.25 NaH_2_PO_4_, 25 NaHCO_3_, 25 glucose, 75 sucrose, and 0.5 ascorbate. Slices were incubated for at least one hour at room temperature before electrophysiological recording in oxygenated artificial cerebrospinal fluid (aCSF) containing (in mM): 124 NaCl, 3 KCl, 1.5 MgSO_4_, 2.4 CaCl_2_, 1.2 NaH_2_PO_4_, 25 NaHCO_3_, and 10-D glucose (for detailed methods see [33,34]). 

#### 2.4.2. Extracellular Field Recordings

Slices were transferred to a recording chamber submerged in ACSF bubbled with 95% O_2_/5% CO_2_ at 30 °C after at least 1 h of incubation. Field excitatory postsynaptic potentials (fEPSP) were recorded from hippocampal Schaffer collateral pathway. CA3 region was stimulated with a bipolar electrode, and the recording electrode was placed in CA1. Presynaptic fiber volley (FV) and fEPSPs were recorded in response to increasing stimulus intensity. The amplitude of FV was measured to determine whether there was any change in the number of presynaptic axons recruited in PCE animals [33]. Basal synaptic transmission was measured as the slope of fEPSPs and plotted as a function of FV amplitude. For paired-pulse ratio (PPR) and LTP experiments, current intensity was set to elicit fEPSPs with the slope equal to 50% of maximum fEPSP. Interpulse intervals were set to 20, 50, 100, 150, and 200 ms in PPR experiments. In LTP experiments, after 10 min of stable baseline recording, induction was initiated by using theta-burst stimulation (TBS) [30,35]. Five TBS sweeps were applied an inter-TBS interval of 20 s. For LTD recording, induction was given using two low-frequency stimulations (LFS: 900 pulses at 1 Hz) delivered at 10 min intervals, preceded by 10 min of stable baseline. Stimulation intensity was set at 60% (during LFS) or 40% (all other times excluding LFS) of the maximum amplitude. LTP and LTD were measured as an average of fEPSP slopes from 50–60 min post induction. Field potentials were recorded using LTP 2.0 software with Axoclamp 2B and analyzed using WinLTP 2.0 software.

Rescue experiments were performed utilizing PSA in the form of colominic acid (CA), a linear polysaccharide containing α-2,8-linked sialic acid residues derived from E. coli (Sigma Aldrich). CA was continuously perfused into the aCSF for 20 min before induction. A dose of 6 μM was used based on our prior work demonstrating this dose can restore LTP deficits in NCAM-deficient mice [19]. 

### 2.5. Preparations of Synaptosomes and Single-Channel Electrophysiology

Synaptosomes were prepared from male pups as previously described [36,37]. Hippocampi were dissected out and homogenized in modified Krebs buffer (mKREBS) consisting of (mM): 118.5 NaCl, 4.7 KCl, 1.18 MgSO_4_, 2.5 CaCl_2_, 1.18 KH_2_PO_4_, 24.9 NaHCO_3_, 10 dextrose, and 10 mg/mL adenosine deaminase. The buffer was supplemented with 0.01 mg/mL leupeptin, 0.005 mg/mL pepstatin A, 0.10 mg/mL aprotinin, and 5 mM benzamide to minimize proteolysis. The homogenate was filtered and centrifuged. Supernatant was removed, and the pellets containing synaptosomes were resuspended in mKREBS buffer [35].

Incorporation of NMDARs from synaptosomal fractions in artificial lipid bilayers was carried out using the “tip-dip” method [37,38]. A thin phospholipid bilayer was formed at the tip of a borosilicate glass pipette (100 MΩ resistance) filled with artificial intracellular fluid (aICF (in mM); 110 KCl, 4 NaCl, 2 NaHCO_3_, 1 MgCl_2_, 0.1 CaCl_2_, and 2 3-N-Morpholino propanesulfonic acid (MOPS); pH 7.4). The synthetic phospholipid was prepared by dissolving 1,2-diphytanoyl-sn-glycero-3-phosphocholine (Avanti Polar-Lipids Inc., Alabaster, AL, USA) in anhydrous hexane (Sigma-Aldrich) at a concentration of 1 mg/mL. A total of 3–5 µL of the synthetic phospholipid was delivered into 500 µL of artificial extracellular bath solution (aECFin (mM); 125 NaCl, 5 KCl, 1.25 NaH_2_PO_4_, 5 Tris HCl, and 0.001 glycine) containing pharmacologic inhibitors for each ion channel subtype, for which recording was not desired (1 µM TTX, 2 µM TEA, 10 µM DNQX, 10 µM UBP302, and 50 µM picrotoxin to block sodium, potassium, AMPA, kainate, and GABA ion channels, respectively). After forming a stable membrane, 2–4 µL of the synaptosome suspension was mixed with 3–6 µL of phospholipid and delivered to the bath solution. Incorporation of receptors into the bilayer was facilitated by gentle stirring and application of voltage across the membrane. Single-channel NMDAR currents were elicited by application of 3 μM glutamate and 1 µM glycine and recorded at holding voltages that elicited visible activity with acceptable resolution between open and closed states while maintaining lipid bilayer stability, averaging ±65 mV. Single-channel currents were low-pass filtered (2 kHz), digitized (5 kHz) (DigiData 1440B, Molecular Devices, San Jose, CA, USA), and acquired with pClamp10 11.2 software (Molecular Devices). Calculation of single-channel open probability (Po) was performed using areas under the current–amplitude histogram fits. Burst analysis was performed on long traces with observed burst activity, and the burst delimiter was defined by an apparent interval between visible bursts for each unique receptor. 

### 2.6. Immunoblotting 

Hippocampal tissue from male pups was homogenized in RIPA buffer (Thermo Fisher Scientific, Waltham, MA, USA) and centrifuged. The supernatant was collected, and protein concentration was measured by the BCA assay kit (Pierce BCA Protein Assay Kit, Thermo Fischer Scientific). In total, 20 µg of protein underwent electrophoresis and transferred to PVDF membranes. Membranes were blocked in 5% non-fat dry milk in TBST for 3–4 h at room temperature. Prior to antibody incubation, the blots were cut into thin strips based on the molecular weight of the probed protein (visualized by Precision Plus Protein™ All Blue Prestained Protein Standards—BioRad, Benicia, CA, USA) to probe for multiple proteins on the same blot and reduce the required incubation volume. The strips were incubated with the specific primary antibody in 5% BSA at 4 °C overnight. After washing three times with TBST, the membranes were incubated with horseradish peroxidase-conjugated secondary antibody (1:3000) for 1.5 h at room temperature. Immunoreactivity was visualized using enhanced chemiluminescence (Thermo Fisher) in a FluorChem Q imager system (Protein Simple). Proteins were normalized to beta actin or GAPDH prior to comparing relative densities. Protein band intensity was quantified by ImageJ 1.53t software. Details of antibodies and dilutions are mentioned in Table 1.

### 2.7. RNA Isolation and Real-Time PCR

A total of 100 µL of hippocampal lysate was mixed well with 1 mL of Trizol reagent (Life Technologies, Carlsbad, CA, USA) and 200 µL of chloroform. The homogenate was then centrifuged, and the upper phase was carefully transferred to a new Eppendorf tube and mixed with an equal volume of 70% ethanol, then loaded in a spin cartridge from PureLink RNA mini kit (Ambion, Naugatuck, CT, USA). RNA concentration was measured using Nanodrop, and 1 µg of RNA was used to make cDNA by Superscript III first strand synthesis system for RT-PCR (Invitrogen, Waltham, MA, USA) according to the manufacturer’s guidelines. Real-time PCR was performed (ABI 7500 Real Time PCR system). GAPDH was used as an endogenous control for normalization. The method of 2^−ΔΔCt^ was used to analyze data.

#### In Vivo Glutamate Recordings

Rats were anesthetized with isoflurane (1–4% inhalation; continuous), previously shown not to alter resting glutamate [39], and placed in a stereotaxic device. To examine changes in glutamate clearance, a ceramic-based microelectrode array (Quanteon, Nicholasville, KY, USA) coated with glutamate oxidase with an attached glass micropipette for drug delivery was inserted into the hippocampal CA1 region (AP: 4.1 mm, ML: 3.5 mm, DV: 3.5) [40,41,42]. To measure glutamate uptake, animals received 1–2 injections at 50 nL increments within a 50–250 nL range of 200 μM glutamate (Sigma-Aldrich) delivered every 2–3 min in one hemisphere. Temporal clearance of glutamate was monitored and expressed as the net area under the curve (AUC). 

### 2.8. Statistical Analysis 

Data analysis was performed using JMP (SAS, Cary, NC, USA), Clampfit 10, and GraphPad PRISM. Unless otherwise indicated, statistical analysis consisted of *t*-tests (paired and unpaired) for bar graphs with two groups, ANOVAs for bar graphs with three groups, or repeated measures ANOVAs for line graphs. Omnibus tests were followed by Tukey’s post hoc. Welch’s *t*-test was used for comparison of open probability and conductance values; Kolmogorov–Smirnov test was used for non-normally distributed data from burst analysis. Results were presented as mean ± SEM, and differences between groups were considered statistically significant at *p* < 0.05.

## 3. Results 

### 3.1. Effects of PCE on Locomotion, Anxiety, and Hippocampal-Dependent Memory

**Figure 2:**
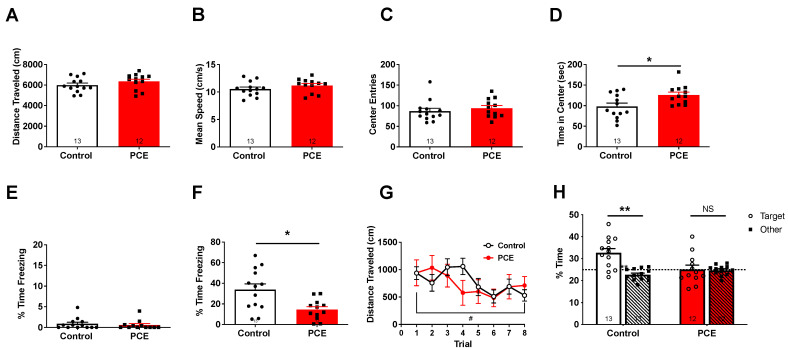
Effects of PCE on locomotion, anxiety, and hippocampal-dependent memory. Total distance traveled (**A**) and mean speed (**B**) was similar between control and PCE animals during the open field task. PCE animals did not differ in entries into the center zone (**C**) but did spend significantly more time in the center zone than controls ((**D**); * *p* < 0.05). While baseline freezing was similar between control and PCE animals during training on day 1 (**E**), PCE animals froze significantly less than controls during the contextual fear test on day 2 ((**F**); * *p* < 0.05). (**G**) PCE animals exhibited slower acquisition in the hidden platform training trials. # *p* < 0.05 for trial 8 versus trial 1 for controls, paired *t*-test. (**H**) Controls exhibited a preference for the target quadrant relative to the average of the other 3 target quadrants (“Other”; ** *p* < 0.01), whereas PCE animals did not (NS = not significant), paired *t*-test. Symbols represent means ± SEM.

To assess general locomotor activity, animals were placed in an open arena and allowed to explore for 10 min. There were no differences in the total distance traveled (*p* = 0.44; Figure 2A) or mean speed (*p* = 0.43; Figure 2B), suggesting PCE did not produce overt alterations in locomotor activity. As rodents will typically spend more time close to the wall, an anxiety-like response referred to as thigmotaxis, as opposed to the unprotected center area [28], we next examined the number of entries into, and time spent in, the center portion of the arena during the open field task to provide an initial screen for anxiety-related behavior [29]. Though PCE only marginally increased the number of entries into the center zone (*p* = 0.07; Figure 2C), the time spent in the center zone was significantly increased in PCE animals compared to controls (*p* = 0.04; Figure 2D), suggesting a reduction in anxiety in animals prenatally exposed to cannabinoid.

We next assessed whether PCE would impair hippocampal-dependent contextual fear memory. Before CFC training, no significant differences in percent time freezing between controls and PCE animals were observed (*p* = 0.91; Figure 2E), indicating similar levels of baseline freezing and activity on day 1. Freezing after the shock on day 1 was also similar between the groups (*p* > 0.05; Both groups froze significantly more on the second day compared to their respective baseline freezing levels (*p*s ≤ 0.001), suggesting learning did occur in both groups. However, PCE animals exhibited a significant decrease in the percent time spent freezing during contextual fear retention compared to control animals (*p* = 0.01; Figure 2F), suggesting an impairment in hippocampal-dependent contextual fear memory. 

To determine if hippocampus-dependent spatial learning and memory were also impaired, the Morris water maze (MWM) was used. While PCE animals did not statistically differ from controls in their acquisition of hidden platform training across the eight training trials (*p* = 0.57; Figure 2G), controls did exhibit a shorter distance on trial 8 versus trial 1 (*p* = 0.02), whereas PCE animals did not (*p* = 0.5), suggesting overall better acquisition in controls. Twenty-four hours after the last training trial, a probe trial was conducted to assess hippocampus-dependent spatial reference memory. While controls exhibited a preference for the target quadrant relative to the other three quadrants (*p* = 0.007; Figure 2H), PCE animals did not (*p* = 0.75), indicating PCE induces deficits in hippocampus-dependent spatial reference memory. 

#### 3.1.1. PCE Impairs Hippocampal Basal Glutamatergic Synaptic Transmission and Synaptic Plasticity

**Figure 3:**
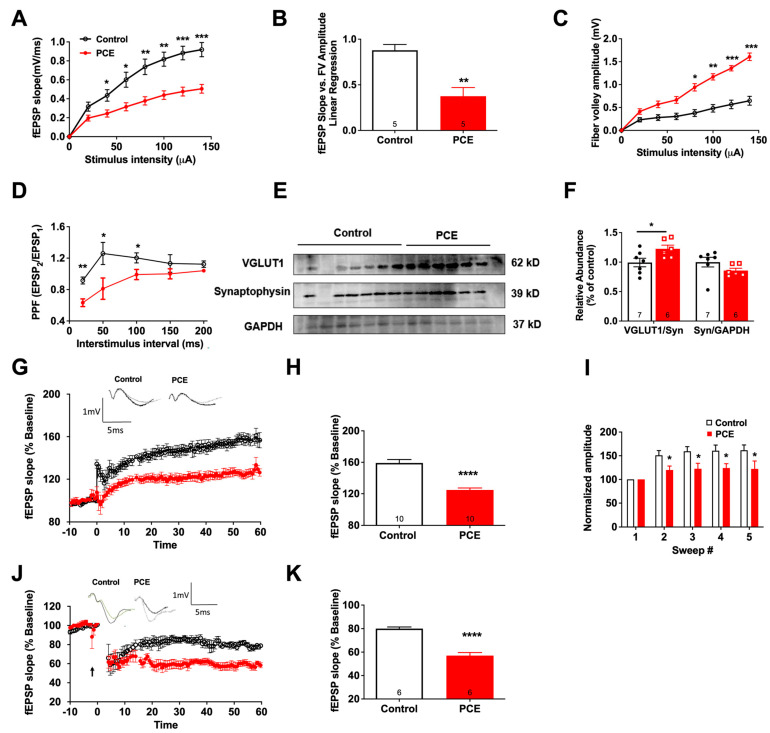
PCE impairs hippocampal basal glutamatergic synaptic transmission and plasticity. (**A**) Input–output curve of fEPSP slope at increasing stimulus intensities showed a significant decrease in basal synaptic transmission in PCE animals. (GABA blockers were not added in our stimulus-response experiments.) (**B**) Slope of the linear regression line of best fit from plotting fEPSP slope vs. FV amplitude showed a significant decrease in the basal synaptic transmission in the PCE animals. (**C**) Fiber volley (FV) amplitude at increasing stimulus intensities showed a significant increase, indicating an increase in presynaptic axonal recruitment in PCE animals. (**D**) Paired-pulse ratio (PPR) expressed in ratio of the fEPSP2 to the fEPSP1 slope plotted as a function of interstimulus interval showed a significant increase in the presynaptic release in PCE animals. (**E**,**F**) Western blot data showed significant increase in VGLUT1 levels in PCE animals, and no significant difference in synaptophysin (Syn) levels between control and PCE animals. (**G**) Long-term potentiation (LTP) graph showing fEPSP slope before and after induction. Mean slope of fEPSPs recorded 10 min prior to theta burst stimulation (TBS) was taken as 100%, and arrow indicates delivery of TBS. fEPSP slope showed a decrease in the PCE animals post TBS. (**H**) LTP bar graph showing fEPSPs recorded for 60 min following TBS induction normalized to baselines levels showed a significant decrease in PCE animals. (**I**) Facilitation of the first fEPSP between trains computed by normalizing the amplitudes of the first fEPSPs for trains #2-5 with the amplitude of first fEPSP of the first train; sweep analysis showed a significant decrease in PCE animals. (**J**) Long-term depression (LTD) graph represents fEPSP slope before and after induction by low-frequency stimulation (LFS). Mean slope of fEPSPs recorded 10 min prior to TBS was taken as 100%, and arrow indicates delivery of LFS; fEPSP slope showed a decrease in the fEPSP slope in the PCE animals. (**K**) LTD bar graph shows fEPSPs recorded for 50–60 min following LFS induction normalized to baselines levels showed a significantly elevated levels of LTD in the PCE animals. Symbols represent means ± SEMs. ns, nonsignificant; * indicates significant difference in control vs. PCE; * *p* < 0.05, ** *p* < 0.01; *** *p* < 0.001, **** *p*< 0.0001.

To determine whether behavioral impairments in the PCE animals are linked to alterations in glutamatergic neurotransmission, we next examined basal synaptic transmission in acute hippocampal slices. The fEPSP slopes were reduced in PCE animals at higher stimulus intensities compared to controls (*p* = 0.001; Figure 3A), suggesting a deficit in baseline synaptic transmission. Next, we measured the fEPSP slopes at various presynaptic FV amplitudes (as a measure of the number of recruited presynaptic fibers), and the FV amplitude and fEPSP slope data were fitted with linear regression [33]. The resulting function, an index of basal synaptic transmission [43], was compared between the groups. PCE animals demonstrated a significant reduction in basal synaptic transmission (*p* = 0.002; Figure 3B).

To determine whether the deficit in basal synaptic transmission in the PCE group was due to alterations in presynaptic axonal recruitment, we analyzed the presynaptic FV amplitude from CA1 synapses at different stimulus intensities. FV amplitudes in PCE animals were higher than those of controls at increasing stimulus intensities (*p* = 0.0005; Figure 3C), suggesting an increase in the recruitment of active afferent axons in the hippocampus in PCE animals [44]. PCE animals also demonstrated a lower PPR value (*p* = 0.02; Figure 3D), indicative of a higher release probability of glutamate. We also observed a significant increase in expression of vesicular glutamate transporter 1 (VGLUT1) in PCE animals (*p* = 0.04; Figure 3E,F), which may have also contributed to the increased glutamate release as observed in by our decrease in PPR in Figure 3C. This increase in VGLUT1 was not due to an increase in presynaptic terminals, as there was no change in synaptophysin levels between the groups (*p* = 0.16; Figure 3E,F). 

The increases in presynaptic function with concomitant decreases in basal synaptic transmission suggest a possible impairment of postsynaptic mechanisms. Therefore, we evaluated synaptic plasticity using acute hippocampal slices to induce long-term potentiation (LTP) or long-term depression (LTD). The fEPSP slope, as a percentage of baseline, showed approximately a 50% decrease in LTP maintenance of PCE animals compared to controls (*p* < 0.0001; Figure 3G,H). When amplitudes of the first fEPSPs elicited by each of five TBSs were normalized to the first fEPSP, each subsequent pulse showed significantly reduced potentiation (*p* = 0.04; Figure 3I), indicating an impairment in LTP induction. In a separate set of experiments, LTD was compared between the groups, and a reduction in the fEPSP slope by >20% from baseline in control slices as compared to a >40% reduction in PCE slices was observed (*p* < 0.0001; Figure 3J,K). Together, these results demonstrate that PCE results in a reduction in LTP and enhancement of LTD in adolescence.

#### 3.1.2. PCE Increases Hippocampal GluN2B Signaling and Decreases NCAM and PSA-NCAM Levels

**Figure 4:**
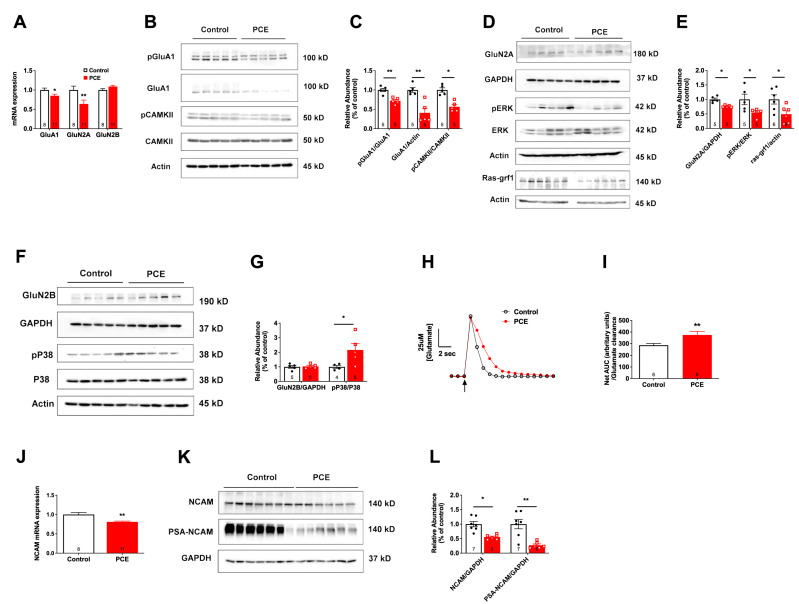
PCE decreases PSA-NCAM levels and alters the balance of signaling between GluN2A- and GluN2B-containing NMDA receptors. (**A**) PCR data revealed a decrease in GluA1 and GluN2A, but no difference in GluN2B mRNA levels. (**B**,**C**) Western blot data revealed a significant reduction in total GluA1, phosphorylation of GluA1 at S831, and phosphorylation of CAMKII in PCE animals. (**D**,**E**) Western blot data indicated significant reductions in GluN2A, phosphorylation of ERK, and Ras-GRF1. (**F**,**G**) Western blot data showed no differences in the GluN2B levels, but an increase in P38 phosphorylation was observed in PCE animals. (**H**) Representative glutamate signals in the CA1 from local application of exogenous glutamate (arrow) in control and PCE offspring. (**I**) Glutamate clearance, expressed as the net area under the curve (AUC), was significantly reduced in PCE animals, indicating reduced glutamate clearance. (**J**) mRNA levels of NCAM were significantly reduced in the hippocampus of PCE animals. (**K**,**L**) Western blot data showed significant reductions in both NCAM and PSA-NCAM levels in PCE hippocampus. Symbols represent means ± SEM. * indicates significant difference in control vs. PCE; * *p* < 0.05; ** *p* < 0.01.

Next, we investigated whether the changes in hippocampal basal glutamatergic neurotransmission were associated with downstream signaling changes in glutamate receptors. We observed a significant reduction in the mRNA level of GluA1 (*p* = 0.04) and GluN2A (*p* = 0.004), but no significant changes in GluN2B mRNA (*p* = 0.07) level (Figure 4A). We also observed a PCE-mediated decrease in both total GluA1 and phosphorylation of GluA1 at Ser831 (*p* = 0.002 and *p* = 0.001, respectively; Figure 4B,C). The downstream phosphorylation of CAMKII was also decreased in PCE animals (*p* = 0.04) but no difference was observed in total CAMKII between the groups (*p* = 0.27). GluN2A receptor levels (*p* = 0.01) as well as the downstream signaling pathway that includes pERK (*p* = 0.04) and Ras-GRF1 (*p* = 0.02) were also significantly decreased in the hippocampus of PCE animals (Figure 4D,E). No difference in the total ERK level was observed between the groups (*p* = 0.67). Although we observed no changes in GluN2B receptor levels (*p* = 0.77) in the hippocampus of PCE animals, increased phosphorylation of P38, which is downstream of GluN2B signaling, was observed (*p* = 0.04; Figure 4F,G), suggesting an increase in extrasynaptic GluN2B-containing NMDA receptor activity. No significant difference (*p* = 0.53) in the total P38 level between the groups has been observed. This could be due, in part, to an increase in extrasynaptic activation and signaling of GluN2B receptors, as PCE animals also exhibited a reduction in the removal of glutamate from the extracellular space (*p* = 0.008, Figure 4H,I). This reduced clearance coupled with the increased glutamate release (as observed in Figure 3D) could lead to synaptic spillover of glutamate and activation of extrasynaptic GluN2B-containing NMDA receptors.

We have previously shown that perturbation in either NCAM expression or polysialylation of NCAM causes an imbalance in signaling between GluN2A- and GluN2B- containing NMDA receptors [18,19,45], leading to deficits in LTP and memory [19], as observed here in PCE animals. Therefore, we next measured the expression of NCAM and PSA-NCAM. We observed a reduction in mRNA level of NCAM (*p* = 0.004; Figure 4J), as well as a significant reduction in NCAM and PSA-NCAM glycoprotein levels (*p* = 0.04 and *p* = 0.001, respectively; Figure 4K,L). Loading controls, GAPDH, and beta actin, did not differ between the groups (ps > 0.05).

#### 3.1.3. PCE Alters Single-Channel Properties of Synaptosomal NMDARs

**Figure 5:**
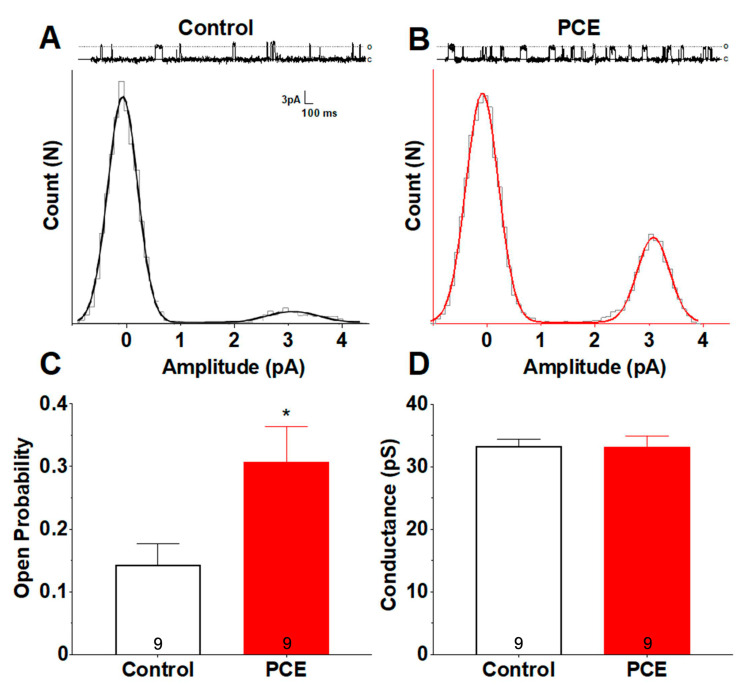
PCE alters single-channel properties of hippocampal synaptosomal NMDARs. Single-channel currents were isolated in the presence of 3 μM glutamate and 1 μM glycine, as well as 1 μM TTX, 2 μM TEA, 10 μM DNQX, 10 μM UBP302, and 50 μM picrotoxin to block sodium, potassium, AMPA, kainate, and GABAA ion channels, respectively. Voltage was clamped to resolve channel openings without compromising artificial lipid bilayer integrity (average ±65 mV; representative traces +80 mV). (**A**,**B**) Amplitude histogram fits and their respective traces from single NMDARs of control and PCE animals. Upward deflections in the current traces represent channel openings. Histogram peaks correspond to primary closed and open states of each receptor, 0 pA and 3 pA, respectively. The difference in open probability is demonstrated by both the greater number of channel openings within the PCE trace compared to control, as well as the larger AUC corresponding to the primary open amplitude of the PCE amplitude histogram. (**C**) Comparison of mean open probabilities between control and PCE revealed an increase in PCE animals. (**D**) Comparison of conductance from isolated control and PCE NMDARs indicated no differences. Symbols represent means ± SEM from n = 9 recordings, n = 5 receptors, and n = 3 animals from the control and PCE groups, respectively. * indicates significant difference in control vs. PCE; * *p* < 0.05.

Alterations in single-channel activity could result in alterations in overall synaptic currents and LTP [46]. As we observed changes in the LTP induction and maintenance, in the next set of experiments, we investigated the single-channel activities of synaptosomal NMDARs. Examination of single-channel traces from controls (Figure 5A) and PCE (Figure 5B) animals revealed an increase in mean channel open probability (Po) in PCE animals (*p* = 0.03; Figure 5C) but no change in ion channel conductance (*p* = 0.92; Figure 5D). These suggest that alterations in single-channel activities of NMDARs may mediate the synaptic plasticity deficits observed in PCE animals.

#### 3.1.4. PCE Alters Single-Channel Burst Properties of Synaptosomal NMDARs 

**Figure 6:**
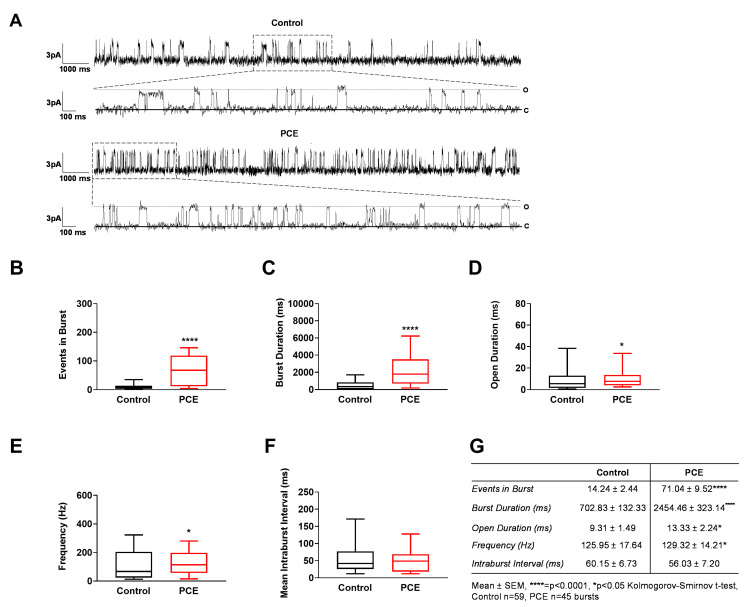
PCE alters single-channel burst properties of synaptosomal NMDARs. Bursts of channel activity were identified via visual inspection of recordings from single receptors. Burst delimiter was set based on the apparent length of closures between long bursts of single-channel activity as denoted on sample traces. (**A**) Comparison of control versus PCE burst activity. The top trace under each group is a 16,000 ms segment to represent long intervals used for burst analysis, whereas the bottom trace in each group is an expanded 3000 ms interval from within the top trace. The large dash boxes and lines indicate the location of the expanded trace within the compressed trace. Solid lines at the bottom of each trace represent closed state and dotted line at the top of each trace represents opened state. (**B**–**F**) Graphical representations of collective means ± SEM for events in burst, burst duration, open duration, frequency, and mean intraburst interval, respectively, as well as the individual values for each parameter from each burst was analyzed. PCE significantly increased the number of events within a given burst, the duration of bursts, the open duration of events within bursts, and the frequency of burst events, whereas having no effect on the mean intraburst interval. (**G**) Table of mean ± SEM values graphed in (**B**–**F**). n symbols represent means ± SEM from n = 59,5 bursts, n = 6,9 recordings, n = 4,5 receptors, and n = 2,3 animals from Control and PCE groups, respectively. **** *p* < 0.0001, * *p* < 0.05.

We next examined the single-channel burst properties from synaptosomal NMDARs (Figure 6A). In PCE animals, the number of events within each burst increased (*p* < 0.0001; Figure 6B) and persisted for longer durations (*p* < 0.0001; Figure 6C) than control receptors. Additionally, open duration within bursts of activity was increased (KSD = 0.29, *p* = 0.02; Figure 6D) and bursts occurred more frequently (*p* = 0.02; Figure 6E) in NMDARs from PCE synaptosomes. The mean intra burst interval, defined as the time between events within a burst, did not differ between the groups (*p* = 0.18; Figure 6F). Table of mean ± SEM values from the graph as shown in Figure 6G. Together, these findings suggest that the burst channel properties of NMDARs possibly contributed to the deficits in synaptic plasticity.

#### 3.1.5. Restoration of PSA-NCAM Rescues LTP Deficits in PCE Animals 

**Figure 7:**
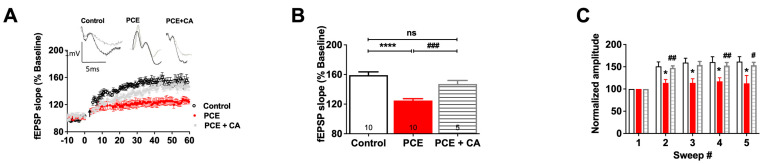
PSA in the form of CA rescues LTP deficits in PCE animals. (**A**) LTP graph showing fEPSP slope before and after induction by TBS. Mean slope of fEPSPs recorded 10 min prior to TBS was taken as 100%, and the arrow indicates delivery of TBS. fEPSP slope showed a reduction in the PCE animals which was increased to control level with the application of CA. (**B**) LTP bar graph showed fEPSPs recorded for 50–60 min following TBS induction normalized to baseline levels. Significant improvement in LTP was observed after CA application as measured by fEPSP slope in PCE animals. (**C**) Within-train facilitation, computed by normalizing the amplitude of the first fEPSPs in trains #2-5 with the amplitude of first fEPSP in the first train. This showed a significant reduction in the sweep in PCE animals, which was increased to control level with the application of CA. Symbols represent means ± SEMs from five rats per group. * indicates significant difference in control vs. PCE; #indicates significant difference in PCE vs. PCE + CA; */# *p* < 0.05, ## *p* < 0.01, ### *p* < 0.001, **** *p* < 0.0001; “ns” indicates not significant.

We have previously demonstrated that PSA in the form of colominic acid (CA) can influence AMPA [47] and NMDAR function [18], as well as LTP [19]. Based on our prior publications [18,19,45], we chose the dose of CA (6 μM) that could potentially restore LTP in PCE animals without altering basal synaptic transmission or LTP in control animals [19,48]. The average EPSP slope after exogenous CA application in the slices of PCE animals was significantly increased 50–60 min after TBS (*p* < 0.001; Figure 7A,B), suggesting that PSA can restore LTP in PCE animals. To investigate whether this increase in LTP was due to synaptic activation of NMDARs, we analyzed the induction data. When amplitudes of the first fEPSPs elicited by each of five TBSs were normalized to the first fEPSP, we observed an increase in the fEPSP amplitude in the PCE animals after CA application (*p* = 0.03; Figure 7C). Together, these results demonstrate that PSA modulates the functionality of NMDA receptors to restore LTP.

## 4. Discussion

The current study demonstrates that PCE resulted in synaptic plasticity and hippocampal-based memory deficits in a rodent model that can persist to the adolescent period of the offspring. These deficits were associated with alterations in glutamate receptor levels and function in the hippocampus along with alterations in their downstream signaling. Furthermore, decreases in hippocampal PSA-NCAM levels were identified as a potential therapeutic target that restored the synaptic plasticity deficits induced by PCE when elevated. We hypothesize that the observed behavioral deficits in our study are due to reduced PSA-NCAM levels, a possibility we will address in future studies.

A dose of 2 mg/kg of WIN55,212-2 was selected based on existing data that 5 mg/kg of Δ9-THC in rats corresponds to moderate exposure of the drug in humans [49,50], and because WIN55,212-2 is estimated to be 3–10 times more potent depending on the outcome measured [22,23], we estimate that a dose of 2 mg/kg corresponds to a moderate exposure in humans [27]. Experimental procedures were performed during the periadolescence and adolescence period (PND 40–65) because most studies examining the consequences of PCE in humans have focused on this period [51,52] thereby allowing for comparison of our results to those obtained in humans. Moreover, adolescent success is highly predictive of adulthood outcomes [53,54] so deficits during this period are likely to produce long-lasting consequences even if neurological alterations associated with PCE do not persist into adulthood. We selected male mice because prior studies indicated that synaptic plasticity [55] and hippocampal function in males [56,57,58], but not females [55,58], were altered by PCE. Likewise, previous studies suggest PCE impairs spatial memory in males [58,59] but not females [58], though a more recent study revealed a deficit in females [60]. We will address sex as a biological variable in future studies. 

It is also important to consider that the treatment paradigm, which consisted of shipping pregnant dams and performing surgery under isoflurane to implant an osmotic minipump, is likely a stressful set of circumstances that may have exacerbated the effects of PCE. It is also possible that the physiological and molecular measures collected in this study were influenced by the learning-dependent behavioral tests that the animals underwent. However, we did wait at least five days after behavioral testing to perform the physiological and molecular measurements. Previous research has demonstrated an elevation in PSA-NCAM levels 24 h post contextual fear conditioning experiments [61]. Similarly, the Morris water maze task has been associated with increased expression of PSA-NCAM approximately 18–20 h following the experimental procedure [62]. Notably, to the best of our knowledge, existing studies have yet to investigate PSA-NCAM expression levels beyond the 5-day mark following the Morris water maze or fear conditioning experiments. Our future studies will be aimed at addressing this issue. 

In the current study, we observed deficits in both hippocampal-dependent contextual fear memory and spatial learning and reference memory for PCE animals, similar to those previously reported [53,54]. Using the open field test, we also observed no alterations in locomotor activity but an increase in time spent in the center of the open field for PCE animals. The lack of change in locomotor activity in the current study is similar to what has been observed by some laboratories [63] but differs from others [59] that have found an increase in locomotor activity in PCE-exposed animals. Likewise, while we observed PCE animals to spend more time in the center of the open field, others have found no differences [63]. Variations in outcome may be related to the cannabinoid used, route of administration, and timing of developmental exposure and/or testing.

To identify potential mechanisms for the observed memory deficits in PCE animals, we compared pre- and post-synaptic glutamate transmission. We found that PCE increased presynaptic glutamate release, which may have been due to increased availability of the glutamate-filled vesicles in the presynaptic terminal of the PCE offspring [64,65]. This was further validated by our findings of an increase in VGLUT1 levels in PCE animals. Though this increase in glutamate release may seem contradictory, given the decreased basal synaptic transmission observed in PCE animals, this reduction in basal synaptic transmission may be related to a possible increase in glutamate binding to synaptic AMPA receptors or the observed decrease in postsynaptic AMPA receptor levels. Hence, it is possible that saturation of synaptic AMPA receptors by excessive extracellular glutamate from either increased presynaptic release, decreased clearance from the extracellular space, or both could have resulted in desensitization of synaptic AMPA receptors, ultimately leading to reduced basal synaptic transmission [66,67,68]. 

Altered basal synaptic transmission and presynaptic release probability in PCE offspring also likely contributed to synaptic plasticity deficits of PCE animals. LTP induction requires concurrent activation of the glutamatergic receptors of both AMPA and NMDA subtypes [69,70], whereas AMPAR expression and trafficking are important for the maintenance of LTP [71]. We observed a decrease in total GluA1 level, as well as reduced phosphorylation of GluA1 at serine 831, which are needed for both LTP and LTD [72]. As larger membrane depolarization leads to increased activation of NMDARs during LTP induction [73], decreased LTP induction in PCE offspring indicates reduced activation of NMDARS, particularly synaptic GluN2A-containing NMDARs [74,75,76]. This is validated by our findings of reduced hippocampal GluN2A levels and downstream signaling (Ras-GRF1 and pERK) in PCE animals. This may also explain the deficits in LTP maintenance, as phosphorylation of ERK is necessary for the LTP maintenance [77]. 

The reduction in Ras-GRF1 levels and the decrease in GluA1 surface receptors might also be due to excessive activation of extrasynaptic NMDARs, which are predominantly GluN2B-containing receptors [78]. Moreover, the reduction in glutamate uptake/clearance observed in PCE offspring can result in glutamate spillover, causing hyperactivation of extrasynaptic GluN2B-containing receptors [79,80]. While we did not observe a significant change in GluN2B receptor levels, we did observe an increase in P38 activation, which is downstream of extrasynaptic GluN2B-containing receptors and suggests a possible increase in extrasynaptic GluN2B signaling. As LTD induction is similarly dependent on the balance between synaptic and extrasynaptic NMDARs [81,82,83], our findings of altered P38 signaling also provide a potential mechanism for the elevated LTD observed in PCE animals. As the subunit composition is well established to alter the properties of individual NMDARs [84], the reduced levels of GluN2A, as well as the reduced activation of GluN2A in our LTP induction analysis, suggest that the observed differences in single-channel properties are also due to altered subunit composition in PCE animals. Together, these findings suggest a possible imbalance in GluN2A- and GluN2B- signaling in PCE animals, resulting in synaptic plasticity and memory deficits, as we have previously shown in other models [18,45,85].

Although we did not examine CB_1_R levels, the increase in glutamate release we observed could be due to altered activation or levels of CB_1_R. CB_1_Rs are more abundant in GABAergic neurons than glutamatergic neurons [86], and activation of presynaptic CB_1_R inhibits GABA release [87] and GABA uptake [56]. As GABA exerts an inhibitory effect [25], PCE could increase glutamate release by reducing GABA release. Our future studies will examine the effects of PCE on GABA-mediated neurotransmission, as well as effects on the balance of excitation and inhibition. 

Prior studies have demonstrated that PCE causes a CB_1_R-dependent increase in cortical glutamatergic neurotransmission [88]. This is in line with our finding of increased presynaptic axonal recruitment in the hippocampus of PCE animals. Increased extracellular glutamate also saturates post-synaptic AMPA receptors, thereby causing reduced frequency and amplitude of AMPAR quantal EPSC [89]. This reduction in AMPAR function can, in turn, stimulate endocannabinoid release [90], resulting in increased in CB_1_R activation [91,92]. Our futures studies will examine how CB_1_Rs modulate neurotransmission in PCE animals. We will also investigate sex-related differences, as prior studies have indicated that PCE impairs cortical [55] and hippocampal [58] synaptic plasticity, as well as metabotropic glutamatergic signaling [14], to alter behavioral outcomes in a sex-dependent manner. Notably, whether female offspring also exhibit a decrease in PSA-NCAM as observed here in males, as well as whether administration of exogenous PSA can rescue PCE-induced impairments in females, remain unknown. 

In conclusion, we postulate that PCE caused alterations in glutamate-mediated neurotransmission via reduced activity of GluA1 and GluN2A and increased signaling through GluN2B, ultimately leading to impaired synaptic plasticity and memory deficits. As NCAM and PSA-NCAM are implicated in the maintenance of synaptic plasticity and memory [93,94], with PSA-NCAM suppressing extrasynaptic GluN2B activity [18,45], we hypothesize that the reduction in hippocampal NCAM and PSA-NCAM levels may mediate the deficits observed in PCE offspring. An imbalance in synaptic versus extrasynaptic signaling was reported to result in shutdown of CREB signaling and hence to reduce expression of downstream genes, including GluN2A [48]. This model is supported by our findings that exogenous application of bacterially produced PSA rescued LTP deficits in PCE offspring, suggesting that PSA-NCAM represents a potential therapeutic target for the treatment of synaptic and cognitive deficits associated with PCE. A recent study suggests that exogenous short fragments of PSA with the degree of polymerization 10 or 12 are sufficient to compensate for the deficiency in endogenous PSA [48]. Additional studies are needed to establish a causal link between the reduction in PSA-NCAM and synaptic and behavioral deficits in PCE. 

## Figures and Tables

**Table 1:** Summary of antibodies and working conditions used in the experiments.

Antibodies	Host & Type	Specificity	Source	Catalog #	RRID	Dilution
Primary Antibodies						
GAPDH	mouse monoclonal	H M Pg Rb R C Dg	EMD Millipore	CB 1001	AB_1078991	1:10,000
β-actin	Rabbit, monoclonal	H M R Mk Dm Z	Cell Signaling Technology	8457	AB_10950489	1:1000
GluA1	Rabbit, monoclonal	M R	Cell Signaling Technology	13185	AB_2732897	1:1000
GluA1 Ser 831	Rabbit, monoclonal	H M	Cell Signaling Technology	75574	AB_2799873	1:1000
CaMKII	Rabbit, monoclonal	H M R	Cell Signaling Technology	11945	AB_2797775	1:1000
Phospho-CaMKII	Rabbit, monoclonal	H M R Dr	EMD Millipore	AB3865	AB_11212950	1:1000
Synaptophysin	Rabbit, monoclonal	H M R	Cell Signaling Technology	36406	AB_2799098	1:1000
VGLUT1	Rabbit, monoclonal	M R	Cell Signaling Technology	12331	AB_2797887	1:500
GluN2A	Rabbit, monoclonal	M R	Cell Signaling Technology	4205	AB_2112295	1:1000
ERK1/2	Rabbit, monoclonal	H M R Hm Mk Mi Dm Z B Dg Pg Ce	Cell Signaling Technology	4695	AB_390779	1:1000
Phospho-ERK1/2	Rabbit, monoclonal	H M R Hm Mk Mi Dm Z B Dg Pg Sc	Cell Signaling Technology	4370	AB_2315112	1:1000
Ras-GRF1	Mouse, monoclonal		Santa Cruz	377234	AB_632334	1:700
GluN2B	Rabbit, monoclonal	H M R	Cell Signaling Technology	4207	AB_1264223	1:1000
P38 MAPK	Rabbit, monoclonal	H M R Hm Mk B Pg	Cell Signaling Technology	8690	AB_10999090	1:1000
Phospho-P38 MAPK	Rabbit, monoclonal	H M R Mk Dm Pg Sc	Cell Signaling Technology	9211	AB_331641	1:1000
NCAM	Rabbit, polyclonal	Ch, H, M, R	EMD Millipore	5032	AB_2291692	1:700
Secondary Antibody						
Anti-rabbit IgG	Goat	R	Cell Signaling Technology	7074	AB_2099233	1:5000
Anti-mouse IgG	N/A	M	Santa Cruz	516102	AB_2687626	1:2000

H—Human, M—Mouse, R—Rat, Hm—Hamster, Mk—Monkey, Mi—Mink, C—Chicken, Dm—D. melanogaster, X—Xenopus, Z—Zebrafish, B—Bovine Dg—Dog, Pg—Pig, Sc—S. cerevisiae, Ce—C. elegans, Hr—Horse, Dr—Drosophila.

## Data Availability

The data that support the findings of this study are available on request from the corresponding authors.
